# Prenatal and Postnatal Disparities in Very-Preterm Infants in a Study of Infections between 2018–2023 in Southeastern US

**DOI:** 10.3390/tropicalmed9040070

**Published:** 2024-03-28

**Authors:** Robin B. Dail, Kayla C. Everhart, Victor Iskersky, Weili Chang, Kimberley Fisher, Karen Warren, Heidi J. Steflik, James W. Hardin

**Affiliations:** 1 Department of Biobehavioral Health & Nursing Science, University of South Carolina, Columbia, SC 29208, USA; everhakc@email.sc.edu (K.C.E.); kh95@email.sc.edu (K.W.); 2 Department of Neonatology, Prisma Health Midlands, Columbia, SC 29203, USA; victor.iskersky@pediatrix.com; 3 Department of Pediatrics/Neonatology, East Carolina University, Greenville, NC 27834, USA; changw@ecu.edu; 4 Department of Pediatrics/Neonatology, Duke University, Durham, NC 27705, USA; kimberley.fisher@duke.edu; 5 Department of Pediatrics, Medical University of South Carolina, Charleston, SC 29425, USA; steflikh@musc.edu; 6 Department of Epidemiology & Biostatistics, University of South Carolina, Columbia, SC 29208, USA; jhardin@mailbox.sc.edu

**Keywords:** premature infant, infection, disparities, mortality, bacteria

## Abstract

Background: The birthrate of Black preterm (BPT) infants is 65% higher than White preterm (WPT) infants with a BPT mortality that is 2.3 times higher. The incidence of culture-positive late-onset sepsis is as high as 41% in very-preterm infants. The main purpose of this study was to examine thermal gradients and the heart rate in relation to the onset of infection. This report presents disparities in very-preterm infection incidence, bacteria, and mortality data amongst BPT and WPT infants. Methods: 367 preterms born at <32 weeks gestational age (GA) between 2019–2023 in five neonatal intensive care units (NICUs) were enrolled to study the onset of infections and dispositions; REDCap data were analyzed for descriptive statistics. Results: The 362 infants for analyses included 227 BPTs (63.7%) and 107 WPTs (29.6%), with 28 infants of other races/ethnicities (Hispanic, Asian, and other), 50.6% female, mean GA of 27.66 weeks, and 985.24 g birthweight. BPT infants averaged 968.56 g at birth (SD 257.50), and 27.68 (SD 2.07) weeks GA, compared to WPT infants with a mean birthweight of 1006.25 g (SD 257.77, *p* = 0.2313) and 27.67 (SD 2.00, *p* = 0.982) weeks GA. Of the 426 episodes of suspected infections evaluated across all the enrolled infants, the incidence of early-onset sepsis (EOS) was 1.9%, with BPT infants having 2.50 times higher odds of EOS than WPT infants (*p* = 0.4130, OR (odds ratio) = 2.50, *p*_or = 0.408). The overall incidence of late-onset sepsis (LOS) was 10.8%, with LOS in 11.9% of BPT infants versus 9.3% (*p* = 0.489, OR = 1.21, *p*_or = 0.637) of WPT infants. BPT infants made up 69.2% of the 39 infants with Gram-positive infections vs. 25.6% for WPT infants; 16 infants had Gram-negative culture-positive infections, with 81.2% being BPT infants versus 18.8% being WPT infants. Of the 27 urinary tract infections, 78% were in BPTs. The necrotizing enterocolitis incidence was 6.9%; the incidence in BPT infants was 7.5% vs. 6.5% in WPT infants. The overall mortality was 8.3%, with BPTs at 8.4% vs. WPT infants at 9.3%, (*p* = 0.6715). Conclusions: BPTs had a higher rate of positive cultures, double the Gram-negative infections, a much higher rate of urinary tract infections, and a higher rate of mortality than their WPT counterparts. This study emphasizes the higher risk of morbidity and mortality for BPTs.

## 1. Introduction

Over 63,000 very-preterm infants, those infants delivered before 32 weeks of gestational age (GA), are born annually in the United States [1]. In 2020, the rate of preterm single births was almost 65% higher for Black mothers compared to White mothers [2]. For every 1000 preterm or low-birthweight (i.e., birthweight < 2500 g) infants born, 25 more Black preterm (BPT) infants die compared to White preterm (WPT) infants [3], and survival without major morbidity is a significant problem [4]. Morbidity such as infection and/or necrotizing enterocolitis (NEC) can lead to extended hospitalizations or even death [5,6]. Moreover, there are significant racial and ethnic disparities in mortality rates. Compared to WPT infants, BPT infants have higher rates of fetal death and infant mortality during the first year of life [3] and, compared to WPT infants, are at a higher risk of all neonatal morbidities [7]. The health of BPT infants is indistinguishably linked to the health of Black women who experience high levels of chronic stress over the life course due to structural racism that disproportionately exposes them to poverty, neighborhood disadvantage, and low educational attainment [8,9]. In a study of over 20,000 mothers and their infants, Black mothers, compared to White mothers, had higher rates of diabetes (including pregestational and gestational), hypertension (including chronic and pregnancy-induced), chorioamnionitis, and premature rupture of membranes (PROM) [10]. Over a decade ago, the annual societal financial burden associated with preterm birth in the United States was found to be at least $26.2 billion [11]. Even though extremely preterm infants (i.e., those born before 28 weeks of GA) account for only 6% of all preterm births, they account for one-third of all costs associated with preterm birth through 7 years of age, which imposes a huge economic burden through early childhood. Black women and their families are placed at a large socioeconomic disadvantage upon the birth of a preterm infant, which may impact the family for generations [12]. Importantly, social determinants of health, or the conditions in which people are born, grow, live, and work, have been linked to the incidence of sepsis in adults and may also be related to sepsis in preterm infants [13]. 

Neonatal sepsis rates range from 1 to 41% in very-preterm infants while they are hospitalized in a neonatal intensive care unit (NICU) [14,15], with the highest rates amongst infants of lower GA and occurrence after one week of age. Infants may be born infected or acquire a nosocomial infection over their NICU hospitalization. Early-onset sepsis (EOS), diagnosed within the first 72 h after birth, is attributed to the infant acquiring a maternal infection when PROM or chorioamnionitis is present [16,17]. EOS confirmed by positive blood cultures is rare with an incidence of 1–4% [18], but EOS is frequently suspected due to the high prevalence of maternal PROM and chorioamnionitis [19], and culture-negative EOS is often treated. Other risk factors for EOS are perinatal asphyxia, amniotic fluid meconium contamination, maternal urinary tract infection, and vaginal examinations [20]. Most often, Group B *Streptococcus* (GBS) and *Escherichia coli* (*E. coli*) are the bacteria associated with EOS. If the mother presents with signs of infection, or PROM, she is treated with antibiotics prior to delivery if there is time to do so before delivery, and the infant is treated with broad-spectrum antibiotics for 24–48 h to monitor blood cultures for bacterial growth, if present.

Late-onset sepsis (LOS) is usually diagnosed after the preterm infant reaches 72 h of age, with the highest incidence between 10–22 days of life [21]. The incidence of LOS is highest for very-preterm or very-low-birth-weight infants and ranges between 8.9–50% [14,15,22]. Some of the common pathogens leading to a diagnosis of LOS are coagulase-negative *Staphylococcus* (CONS) [22,23] in 29–50% of Gram-positive cultures, *Staphylococcus aureus* (4–23%), and *Enterococcus* species (3–16%) [15,24]. The usual pathogens associated with Gram-negative sepsis in very-preterm infants are *Escherichia coli* (3–13%), *Klebsiella* spp. (4–8%), *Pseudomonas* spp. (2–5%), *Enterobacter* spp. (2.5–21%), *Serratia* (0.8–2%), and *Acinetobacter* (0.1–2%) [15,24]. These types of infections can be frequent and occur several times during a NICU hospitalization, due to the vulnerability of preterm infants with weakened immune systems, and the frequency of contact with multiple hospital personnel, indwelling central lines, and respiratory treatment with apparatus that requires warming through fluids [16]. Signs of infection are often ambiguous and may include apnea and bradycardia, respiratory distress, thermal instability, feeding intolerance, lethargy, or the infant clinically not at baseline per staff [16]. Evaluations to rule out and treat based on the suspicion of LOS are less standardized than when the mother’s clinical obstetrical history prompts the clinician to rule out and conduct surveillance treatment for EOS, especially as clinicians attempt to scale back the overuse of antibiotics [16]. 

LOS leads to increased morbidity and mortality, with the incidence increasing as the GA at birth decreases [24]. It is imperative that researchers examine health disparities in the maternal–infant population so etiological pathways can be exposed, and potential interventions developed to lessen those disparities. This analysis aimed to explore disparities between Black and White mothers’ prenatal health from available data, with a focused examination of disparities between Black and White preterms with regard to morbidities including EOS, LOS, NEC, and bacteria present in the urinary tract, respiratory tract, and cerebral spinal fluid (CSF), as well as infant mortality between birth and NICU discharge/transfer/death.

## 2. Materials and Methods

This study (protocol 00082482) was approved by the Medical University of South Carolina Institutional Review Board (IRB) with a Federal Wide Assurance #1888, as a multi-site study using a single IRB, and study procedures and documents were site-approved by local site IRBs. The study is funded through National Institute of Health, and National Institute of Nursing Research (R01NR017872), beginning in 2018 through 2023. A detailed study protocol [25] is published. A potential participant mother consented for herself and her infant’s participation in the study prior to the infant reaching 6 h of age. Mothers could also consent for infant participation prior to the infant’s birth, while she was being observed in non-active labor in the hospital. Upon an infant’s birth, an infant could be included in the study if birthweight was 500–1500 g and GA was between 24–31 6/7 days by obstetrical dating noted in the record. Infants must also be born at the hospital of enrollment and not transported in from a referring hospital. Exclusion criteria included any major anatomical or cardiac defect known at birth and any major abdominal defect visible at birth. Five NICUs in North and South Carolina participated in this longitudinal, observational trial. During the pilot phase to test methods, one infant at each NICU was enrolled and not included in analysis because of the need to refine instrumentation for capture of quantitative temperature data. The main purpose of this study was to examine average daily central–peripheral temperature difference (CPTd) values over each infant’s first 28 days to determine if abnormal thermal gradients, or CPTd values of <0 °C and/or >2 °C, were related to the onset of infection [25]. Body temperatures were measured every minute using a thermistor (disposable skin sensor, 499B, Cincinnati Sub Zero, Cincinnati, OH) attached to each infant’s abdominal/flank area, for central temperatures, and one to the foot, for peripheral temperatures. Temperatures were measured, stored, and downloaded from a research data logger (Squirrel SQ2010, Grant Instruments, Cambridge, England). Analyses related to temperature measures and the onset of infection are ongoing. 

Infants were hospitalized in 1 of 5 hospitals, each with a level IV regional NICU servicing many counties from central and eastern North Carolina and central and coastal South Carolina. Each NICU has several attending neonatologists, and all NICUs had care teams consisting of nurses, respiratory therapists, neonatal nurse practitioners, and physicians. All NICUs have standards dictating the use of double-walled incubators to care for their infants, and infants are on cardio-pulmonary monitors. This study enrolled infants beginning in 2019, through the pandemic years of 2020–2021, and completed enrollment in December 2023. Although enrollment greatly decreased during the pandemic, most units continued to enroll infants, and our experiences with this study during the pandemic are published [26].

All clinical context data were entered into a REDCap [27] database developed for this study, which included maternal obstetrical history and delivery details, infant morbidities, medical treatments, feedings, radiology reports, and disposition at discharge, transfer, or death. Maternal obstetrical history related to prenatal care visits was not captured for this study. Morbidity data included any suspected infection, type of cultures obtained, results, and treatment for infection. In all NICUs, standard of care is to respond to signs and symptoms of infection reported by an infant’s bedside nurse with laboratory testing to rule out infection, and, most often, broad-spectrum antibiotics are initiated for at least a 36 h time period while waiting for culture results. Signs that a preterm may have an infection include apnea and bradycardia, feeding intolerance, lethargy, temperature instability including hypothermia, and the need for respiratory support [16].

## 3. Results

Between June 2019 and December 2023, 367 infants met the inclusion criteria and were enrolled for study measures; five of these infants were excluded from analysis as they were enrolled in the pilot phase of the study. The analytic sample totaled 362 infants. These infants were 62.7% Black (n = 227), 29.6% White (n = 107), and the remaining 28 infants were classified as American Indian, Asian, Hispanic, or other. Infants were 50.6% female and ranged between 24–32 weeks GA at birth, with a mean of 27.66 weeks (SD 2.06). Birthweight ranged from 500 to 1490 g, with a mean of 985.24 (SD 260.45) grams. BPT infants averaged 968.56 g at birth (SD 257.50) and 27.68 (SD 2.07) weeks GA compared to WPT infants in this study with a mean birthweight of 1006.25 g (SD 257.77, *p* = 0.2129) and 27.67 (SD 2.00, *p* = 0.9817) weeks GA. In the following subsections, we include the unadjusted *p*-value associated with the two-sample test of proportions (*p*), as well as the odds ratio (OR) and adjusted *p*-value (*p*_or) for race from a logistic regression of the outcome adjusted for GA and birthweight. Odds ratios larger than one indicate a higher likelihood of the outcome for BPTs, and odds ratios smaller than one indicate a reduced likelihood of the outcome for BPTs.

### 3.1. Maternal Morbidities

In this sample, there were no stark differences between the delivery mode with 74.44% of Black mothers and 78.50% (*p* = 0.420, OR = 0.70, *p*_or = 0.224) of White mothers being delivered of their preterm infant by caesarean section. Almost all mothers stated they had received prenatal care prior to the delivery of their infant (97.4% Black women, 97.2% White women, *p* = 0.933, OR = 1.20, *p*_or = 0.806). More Black mothers cultured positive for GBS prior to delivery than White mothers (24.2% vs. 18.7%, *p* = 0.258, OR = 1.45, *p*_or = 0.203) and, similarly, more Black mothers were diagnosed with chorioamnionitis than White mothers (15.1% vs. 10.3%, *p* = 0.235, OR = 1.68, *p*_or = 0.171). However, the incidence of PROM was approximately equal between Black and White mothers (32.2% vs. 33.6%, *p* = 0.787, OR = 1.04, *p*_or = 0.883). 

During pregnancy, Black women had pre-eclampsia less often than White women (31.7% vs. 36.4%, *p* = 0.392, OR = 0.67, *p*_or = 0.135), but had twice the incidence of gestational diabetes compared to White women (7.9% vs. 3.7%, *p* = 0.150, OR = 2.40, *p*_or = 0.125). Black women had a much higher incidence of cardiovascular disease prenatally (14.1% vs. 3.7%, *p* = 0.004, OR = 3.92, *p*_or = 0.013), renal disease (0.9% vs. 0, *p* = 0.329), and a slightly higher incidence of diabetes (8.8% vs. 7.5%, *p* = 0.673, OR = 1.16, *p*_or = 0.730).

### 3.2. Infant Infections

#### 3.2.1. Early-Onset Sepsis

**Figure 1:**
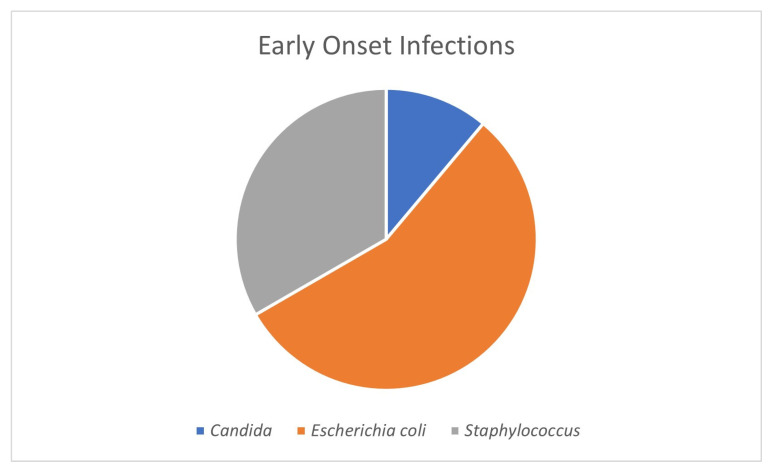
Early-onset infections for 7 infants.

In this sample, there were seven infants with confirmed, culture-positive early-onset infection. Five BPT infants had EOS: three infants with *E. coli*, one infant with *E. coli* and *S. capitus*, and one infant with *S. capitus*; one WPT infant with *S. hominis*. One infant of other race/ethnicity was diagnosed with *Candida.* See Figure 1. The incidence of EOS was 1.9% of the enrolled infants, with BPT infants having a 2.2% incidence and WPT infants having a 0.9% (*p* = 0.4156, OR = 2.50, *p*_or = 0.408) incidence. All infants with EOS had a mean birthweight of 915.29 g and GA of 26.4 weeks. All Gram-negative EOS cases occurred in BPT infants, with one of those infants dying at two days of age (female infant, birthweight of 670 g, and 25 weeks GA).

#### 3.2.2. Late-Onset Sepsis

**Figure 2:**
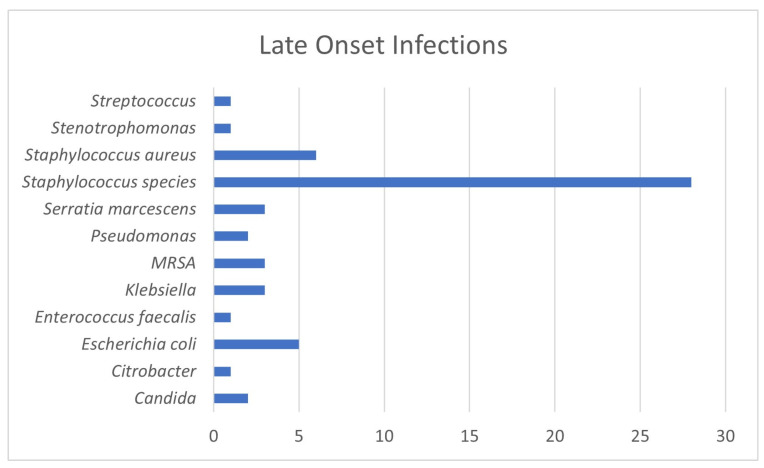
Late-onset infections for 39 infants. *Staphylococcus* species: *S. capitus*, *S. epidermidis*, *S. haemolyticus*, *S. hominis*, normal flora, possible contaminants.

LOS occurred in 10.8% of the enrolled infants, with 39 infants having a positive culture for bacterial or fungal blood infection (see Figure 2) prior to discharge, transfer, or death. In total, there were 54 separate culture-positive infections amongst the 39 infants, with some infants having multiple infections of the blood. LOS occurred in 11.9% of BPT infants versus 9.3% (*p* = 0.4887, OR = 1.21, *p*_or = 0.637) of WPT infants. Infants who acquired LOS were smaller at birth compared to infants who did not acquire LOS, with BPTs’ mean birthweight at 773.44 g versus WPTs’ mean at a 788.2 g average (see Table 1). Their counterparts who did not acquire LOS are as follows: BPTs 996.7 g versus WPT infants 1028.73 g; however, disparities remain between the Black and White infants over these trends (see Table 1). Across 39 infants with Gram-positive infections during their NICU hospitalization, there were 28 instances of *Staphylococcus* (*capitus*, *epidermidis*, *haemolyticus*, and *hominis*) which may be recognized as normal skin flora or deemed to be contaminants. Other Gram-positive LOS cases included one BPT infant with *Enterococcus faecalis*, six cases of *S. Aureus* in five BPT infants and one WPT infant, and one case of *Streptococcus* and one case of Stenotrophomonas, both in BPT infants. Additionally, there were two cases of *Candida*-positive blood cultures, both in BPT infants and both in the same enrollment unit. 

#### 3.2.3. Gram-Positive versus Gram-Negative Infections

Across all EOS and LOS culture-positive infections, 39 infants had culture-positive blood infections with Gram-positive bacteria and 16 infants had culture-positive Gram-negative infections. BPT infants made up 69.2% of infants with Gram-positive infections versus 25.76% for WPT infants. There were 16 Gram-negative culture-positive infections in this cohort, with 81% of the cases identified in BPT infants versus 18.8% in WPT infants (*p* = 0.5368). 

#### 3.2.4. Urinary Tract Infections 

Twenty-three infants, or 6.4% of the sample, had positive urinary tract cultures in this study with 27 bacterial readings provided. This rate of urinary tract infections is similar to that found in other studies [28]. Of the 23 infants having positive urinary tract cultures, 78% were BPT infants. Only four samples identified *Staphylococcus* contaminants: five Gram-negative bacteria including *Citrobacter*, *E. coli*, *Enterobacter*, *Klebsiella*, and *Proteus mirabilis*, as well as *Enterococcus faecalis*, in addition to contaminant *Staphylococcus*. Of note, three infants with urinary tract infections identified also had NEC (two BPT and one WPT infants), and two of those infants, both BPTs, died.

#### 3.2.5. Respiratory Tract Infections

Not all enrollment units use the culturing of tracheal aspirates or nares as surveillance for respiratory tract infections as standard management; however, two NICUs reported one positive respiratory tract culture, one NICU reported three, and one NICU reported 14 positive respiratory cultures. Of the 19 infants with positive cultures, 12 were BPTs, 6 were WPTs, and 1 was other than Black or White. In addition to all bacteria cultured in urinary tract infections (listed above in Section 3.2.4), including *Stenotrophomonas* and *Proteus*, there was one respiratory culture positive for adenovirus.

#### 3.2.6. Cerebral Spinal Fluid Infections

There were two BPT infants diagnosed with positive CSF infections. A 570 g male BPT infant born at 24 weeks GA had a positive culture for *Candida* in the blood on day of life 5, CSF on day of life 7, with death at 12 days of age from multiorgan failure, coagulopathy, and metabolic acidosis due to sepsis. A BPT female infant born at 700 g, 24 weeks GA, had EOS with *E. coli* and a CSF infection with *Paenibacillus* spp. on day of life 2. This infant was discharged home at 5 months of age, with an intraventricular hemorrhage of a left grade II and right grade I, with resolution by 2 months of age.

#### 3.2.7. Necrotizing Enterocolitis

Infants were diagnosed with NEC in four out of five enrolling NICUs; NEC diagnosis was recorded from the EMR if abdominal radiographs specified pneumatosis intestinalis and/or free air in the peritoneum. There were 25 infants given a NEC diagnosis, or a 6.9% incidence, amongst 17 BPT, 7 WPT, and 1 other than Black or White infants. The incidence among BPT infants in this sample was 7.5% versus 6.5% in WPT infants (*p* = 0.7545). Overall, the incidence of mortality amongst the NEC cases was 32%, with a 35.3% mortality in BPT infants and 28.6% in WPTs diagnosed with NEC. Of the NEC cases, seven infants also had a co-occurrence of bacterial infections in blood, two of those infants had positive cultures in blood and urine, and one infant had positive blood and respiratory cultures. These bacteria in the blood included *E. coli*, methicillin-resistant *S. Aureus* (MRSA), *S. aureus*, *Serratia*, *Streptococcus*, and *Candida*, as well as three cultures with *S. capitus*. Urine bacteria included *E. coli*, *Enterococcus faecalis*, and MRSA, and one infant had *Serratia* in the blood and *Serratia*, *S. aureus*, and *Stenotrophomonas* in the respiratory tract. 

#### 3.2.8. Mortality

The overall mortality in this study was 8.3%, with that of BPT infants at 8.4% and WPT infants at 9.3%, (*p* = 0.7676). When mortality is compared within all BPT infants in this study, the incidence of mortality amongst BPT infants with sepsis or NEC (N = 43) was 30.2% compared to 3.3% in BPTs without sepsis or NEC (n = 184). Amongst all WPT infants, the incidence of mortality amongst infants with sepsis or NEC (n = 16) was 12.5% versus 8.8% in those without sepsis or NEC (n = 91). Of the 30 enrolled infants that died while hospitalized in the NICU, the cause of death was listed as infection and/or NEC in 16 infants. Of these 16 infants, 87.5% (n = 14) were BPT infants. Other causes of death were neurological in one BPT and two WPT infants, respiratory failure or related in four BPT and two WPT infants, and cardiac or multiorgan failure in four WPT infants. 

## 4. Discussion

Study results from this observational trial of 362 preterm infants, born at less than 32 weeks GA and at less than 1500 g birthweight, reveal that disparities between NICU course, morbidity, and mortality outcomes continue to exist between Black and White preterm infants. Our enrollment period took 55 months to meet our enrollment goal, due to the existence of the coronavirus disease outbreak in 2019, which caused a global pandemic beginning in March 2020 and into 2023. This study intended to observe infants for the onset of infection, and, although enrollment began in 2019, our study is limited by reduced enrollment during many months and altered infection control procedures across several of our enrollment NICUs put in place due to the pandemic with reduced or no visitation, all of which is outlined in a previously published paper [26]. Our overall incidence of LOS (10.8%) is lower than recent trends [15,29] which may reflect the strict infection control procedures in many of our enrollment units during the pandemic. Masking, increased handwashing, and limited visitation may have decreased the spread of nosocomial infections; however, this hypothesis has been studied and found to have no effect on hospital-acquired infections [30,31] which highlights the need to be continuously vigilant to reduce LOS in preterms. 

Despite enrollment during all of the pandemic months and beyond, our incidence of EOS (1.9%) is only slightly higher than that of studies prior to the pandemic [18] and, like these studies, we found *E. coli* to be the most prevalent cause for EOS. BPT infants had over twice the risk of EOS than their WPT counterparts. Although this comparison did not reach statistical significance, the disparity is clinically significant. EOS is attributed to acquired infection prenatally or during birth, and, in our study, mothers of BPT infants had a 1.45 times increased odds of being positive for Group B *Streptococcus* prior to birth and a 1.68 times increased odds of having suspected chorioamnionitis compared to mothers of WPT infants, which may increase the chance of EOS for these infants. In our study, research nurses documented chorioamnionitis from the EMR. The EMR documentation of chorioamnionitis at each of the five enrollment sites may vary by the criteria used for diagnosing chorioamnionitis, ranging from suspicion due to maternal symptoms including maternal fever, or amniotic fluid that is not clear and/or purulent or foul-smelling [32]. The increased odds of having suspected chorioamnionitis may have led to EOS in the BPT infants rather than the increased odds of maternal diagnosis with Group B *Streptococcus*, as *E. coli* bacteria were more prevalent than Group B *Streptococcus* amongst the cases of EOS. Our study cohort of mothers almost entirely verbalized they had received prenatal care; however, with a range of GA between 24–32 weeks, there is a high possibility that the number of prenatal care visits were low for mothers of earlier GA infants. Without routine prenatal care, mothers cannot be assessed and treated for infection. Future studies should focus on a detailed assessment of prenatal care visits, treatment for any prenatal infections, and the documentation of clinical versus suspected chorioamnionitis with results leading to interventions to reduce the disparities.

Although sepsis rates for very-preterm infants are decreasing, studies are showing faster declines in the rates of sepsis for BPT compared to WPT infants [10]; our study confirms the rates of EOS, LOS, NEC, and morality remain higher, although in not a statistically significant manner, for BPT infants compared to WPT infants, similar to Boghossian et al. [33]. In this sample of very-preterm infants, BPT infants averaged 970.03 g at birth compared to WPT infants with a mean birthweight of 1006.25 g, both groups at a mean of 27.6 weeks GA. Perhaps this difference reflects the impact of social determinants of health effects on the BPT infants’ birthweight compared to the WPT infants. Our enrollment NICUs were all North and South Carolina, evenly divided between urban and rural areas. Investigators have attempted to account for the difference in the preterm birthrate in Black individuals compared to their White counterparts, without definitive pathways [34]. Our enrollment was 62.7% Black versus 29.6% White, in the areas of NC and SC, which are both approximately 60% White individuals versus 25% Black individuals. Researchers also posit that the only plausible explanation for the differences in the preterm birth rate between Black and White individuals is racism, with biology, social determinants, immune response, and stress only contributing to a small portion of the pathogenesis [35]. Diamond-Smith et al. found mothers who are underweight prior to pregnancy have a higher rate of preterm birth and that Black mothers were more likely to be underweight than White mothers [36]. These researchers also found that mothers with public health insurance and an association with WIC had increased odds of being underweight. Additionally, we know that underweight mothers are more likely to have low-birthweight infants [37]. A low birthweight and less-than-optimal weight gain during NICU hospitalization is associated with sepsis and NEC [16,38]. Future studies should examine the aspects of the social determinants of the health of mothers with sepsis outcomes in their infants, with a focus on the disparities between races and ethnicities. Interventions may target increasing access to healthcare, optimizing prenatal diets for mothers to ultimately decrease rates of EOS and LOS in their preterm infants.

With this study, we see differences in the pathogens causing sepsis in BPT and WPT infants. We found that, out of 16 infants with Gram-negative culture-positive infections in this cohort, 81% of the cases were identified in BPT infants versus 18.8% in WPT infants. Even though our study was not powered to find a statistical difference, this disparity is important in that we know that Gram-negative sepsis is associated with significant mortality [39]. Researchers have found non-White race as a risk factor for Gram-negative infections [40]. In studies of central-catheter-associated bloodstream infection events, Black patients are more likely to acquire an infection than White patients [41]. As with other studies [15,42], *E. coli* was the most prevalent type of Gram-negative bacteria associated with LOS, EOS, and one of the bacterial meningitis cases in our cohort. Three of the deaths in this sample were attributed to *E. coli* sepsis by the infants’ attending physicians. Research is showing that a strain of *E. coli* is multi-drug-resistant [43]. Clinicians now must monitor the response to antibiotics and search for the right treatment to resistant *E. coli* infections. Research shows that the proportion of extended-spectrum beta-lactamase (ESBL)-producing strains of *E. coli* is rising to the level of over 60% as recently as 2021 [42].

In our study, there were 28 instances of *Staphylococcus* (*capitus*, *epidermidis*, *haemolyticus*, and *hominis*), which many clinicians deem to be normal skin flora or contaminants. However, in this cohort, four infants with these bacteria in their blood cultures died during this study, and, in two cases, infants only grew *S. capitus* from the blood culture and had NEC diagnosed prior to death. A research team from the United Kingdom conducted a retrospective study to determine if *S. capitus* infections lead to worse outcomes in very-preterm infant but found no differences in mortality or outcomes with *S. epidermidis*, *S. capitus*, *S. haemolyticus*, and *S. warneri* [44]. Researchers have also identified *S. capitis* strains that belong to the NRCS-S clone subgroups, which may lead to virulent LOS and poor outcomes [45]. As in our study, CONs are a prevalent bacteria growing out of blood cultures [46] and, in this study, 10 infants had *S. capitus* identified in their blood cultures; therefore, it may be difficult to discern if the CONs are a contaminant or infection that may lead to poor outcomes. More research is needed to investigate CON infections and their virulence so clinicians can manage this bacterium in the blood of fragile NICU patients effectively. 

This cohort of infants had a 6.4% overall incidence of urinary tract infections, and, interestingly, 78% were BPT infants. Studies suggest a urine culture should be part of workups to evaluate preterms for LOS [47]. Because we did not collect decision data, there is no way of knowing how a decision to obtain a urine culture was made when an infant was showing signs and symptoms of sepsis. More studies should include an examination of what is included in a workup for infection, how decisions are made as to which cultures are obtained, and the methods for obtaining cultures. A limitation of this study is the lack of standardized procedure for obtaining urine cultures amongst the five enrollment sites. NICU clinicians have obtained urine cultures by sterile catheterization, suprapubic aspiration, or a topical specimen-bag catchment system [48], And, many times, all three procedures may be used in one NICU depending on the ordering clinician, nurse’s skills, or infant size. Our study data collection did not ascertain the method of urine specimen for cultures obtained. Urinary tract infections may be an important etiology of sepsis or NEC; therefore, more research is needed to examine the entry point of bacteria and the etiological pathways to infection in preterm infants. Researchers have identified the most common bacterial pathogens as *Enterococcus* spp. (20%), *E. coli* (19%), and *Klebsiella* spp. (18%) in the urinary tract [28]. In our study, *Proteous mirabilis* caused a urinary tract infection in a 624 g, male WPT infant, and a concordant respiratory tract infection. This Gram-negative bacteria can be found in the human intestines and is associated with urinary stones [49]. One urinary tract infection cultured *mycoplasma* and *ureaplasma*, which are bacteria common to the genital tract; *ureaplasma* was also identified in one respiratory tract infection. Researchers have linked *ureaplasma* with an increased incidence of bronchopulmonary dysplasia (BPD) [50]. 

In our study, local NICU standards dictated whether to culture tracheal aspirates. This practice has been debated for many years. The practice amongst 4 of 5 NICUs in our study yielded 19 positive cultures, with nine Gram-negative pathogens. There was one 81 -g, 26-week GA, WPT male infant whose culture grew *Serratia*, *S. aureus*, and *Stenotrophomonas*, and developed NEC. Studies have shown an association between Gram-negative bacteria in the tracheal aspirate and developing severe BPD in infants of less than 26 weeks GA [51]. More research is needed to determine if respiratory cultures or tracheal aspirate cultures should and can be used in the medical management of very-preterm infants.

This sample had one case where a BPT female infant born at 700 g was found to have *Paenibacillus* meningitis on the second day of life, and, yet, this infant went on to recover and was discharged home at 5 months of age. A review revealed *Paenibacillus* sepsis and meningitis to be rare in very-preterm infants, and most cases reported lead to increased mortality [52,53]. Of note, this BPT female had an EOS with *E. coli* as well, for which she was treated with ampicillin, gentamicin, and erythromycin. Two days later, cefotaxime was added to the treatment. Three days later, when *Paenicbacillus* spp. was cultured from the CSF, antibiotics were changed to gentamicin and cefotaxime, with gentamicin discontinued the next day. The infant was treated with additional antibiotics ending at 3 weeks of age with no other incidence of infection.

The incidence of NEC in this study was within the current prevalence at 6.9% [54]. As with other studies [55], we found racial disparities amongst our NEC cases, with BPT infants having a higher incidence of NEC at 7.5% versus WPT infants at 6.5% (*p* = 0.7545). Even though statistical significance was not found between this comparison, the finding is suggestive of areas for further research. Researchers have found that a smaller birthweight is a risk factor for NEC [56]. BPT infants in our study averaged a lower birthweight compared to WPT infant, with approximately the same mean GA. Other possible causes include social, cultural, education, economic, and environmental factors, according to Cuna et al. [55]. Our study was not constructed to evaluate factors for social determinants of health; however, future research could examine the interplay between social determinants and infections/NEC outcomes by race.

An important finding exposed concerning maternal disparities was the significant difference between prenatal cardiovascular disease in Black women who gave birth to preterm infants compared to White women. The incidence of concomitant cardiovascular disease and pregnancy is high, with Petersen et al. finding 66% of White and 11% of Black women with this co-occurrence; however, more Black mothers have obstetric complications and fetal events compared to White mothers (44% versus 33%), with heart failure significantly higher for Black mothers [57]. Other researchers have found mothers with a history of chronic hypertension and hypertensive disorders of pregnancy have a higher odds for the risk of preterm birth [58]. More research needs to focus on the increased incidence of preterm birth and morbidities in BPT infants in relation to maternal cardiovascular disease including hypertension. 

## 5. Conclusions

In our study of 362 very-preterm infants aimed at examining thermal gradients and heart rates in relation to the onset of infection, we conducted an analysis to explore the disparities between the incidence of infections, NEC, and mortality between Black and White very-preterm infants. Although this analysis was not the main aim of this five-year, multi-site study in NICUs in North and South Carolina and was not powered for these multiple analyses, we highlighted existing racial disparities in infection outcomes, NEC, and mortality, which have clinical and social importance, as well as give direction for future research to decrease disparities amongst very-preterm infants. The study confirms the incidence within published rates for EOS, LOS, and NEC with bacteria prevalent from the blood, urinary tract, trachea, and CSF comparable with studies across the United States. BPT infants in this study dealt with many more Gram-negative infections than their WPT infant peers, with lower body weights. Importantly, BPT infants who had sepsis or NEC had a mortality rate of 30.2% compared to WPT infants at 12.5%. There is an urgent need for research that focuses on mortality and morbidity disparities related to infections in very-preterm infants with adequate samples to determine pathways for interventions to decrease the disparities. 

## Figures and Tables

**Table 1:** Demographics for Black and White preterms with positive blood cultures. Key: BW: birthweight in grams; SD: standard deviation; GA: gestational age in weeks.

Positive Blood Cultures	Black Preterms					White Preterms				
	N	BW Mean	BW SD	GA Mean	GA SD	N	BW Mean	BW SD	GA Mean	GA SD
Early Onset Sepsis (7)	5	1002.4	340.22	26.38	2.28	1	745	NA	26	NA
Late Onset Sepsis (39)	27	773.44	200.54	26.26	1.99	10	788.2	178.5	26.2	1.48
Infants without LOS (323)	199	996.7	252.69	27.87	2.02	97	1028.73	254.79	27.81	2.01
Gram Positive Bacteria (35)	22	828.67	226.14	26.33	1.17	8	787.67	188.48	26.22	1.56
Gram Negative Bacteria (16)	9	747.62	229.24	25.92	2.47	3	767.67	43.06	25.67	0.58

## Data Availability

The data are being analyzed. Please contact Robin B. Dail, at rdail@mailbox.sc.edu to inquire about data access.
